# Protocol for separating cancer cell subpopulations by metabolic activity using flow cytometry

**DOI:** 10.1016/j.xpro.2024.103105

**Published:** 2024-06-01

**Authors:** Wiktoria Blaszczak, Bobby White, Pawel Swietach

**Affiliations:** 1 Department of Physiology, Anatomy and Genetics, University of Oxford, Parks Road, OX1 3PT Oxford, UK

**Keywords:** Flow Cytometry, Cancer, Metabolism

## Abstract

Cells, even from the same line, can maintain heterogeneity in metabolic activity. Here, we present a protocol, adapted for fluorescence-activated cell sorting (FACS), that separates resuspended cells according to their metabolic rate. We describe steps for driving lactate efflux, which produces an alkaline transient proportional to fermentative rate. This pH signature, measured using pH-sensitive dyes, identifies cells with the highest metabolic rate. We then describe a fluorimetric assay of oxygen consumption and acid production to confirm the metabolic contrast between subpopulations.

For complete details on the use and execution of this protocol, please refer to Blaszczak et al.^1^

## Highlights

• Metabolic activity is a key cellular phenotype, determined by a myriad of variables
• Cancer cells with higher lactic acid permeability tend to have higher metabolic rate
• Lactate unloading produces alkaline transients that identify cells by metabolic rate
• Implementation for FACS generates subpopulations of distinct metabolic phenotype

## Abstract (editor highlights)

Publisher’s note: Undertaking any experimental protocol requires adherence to local institutional guidelines for laboratory safety and ethics.

## Graphical abstract

**Figure:**
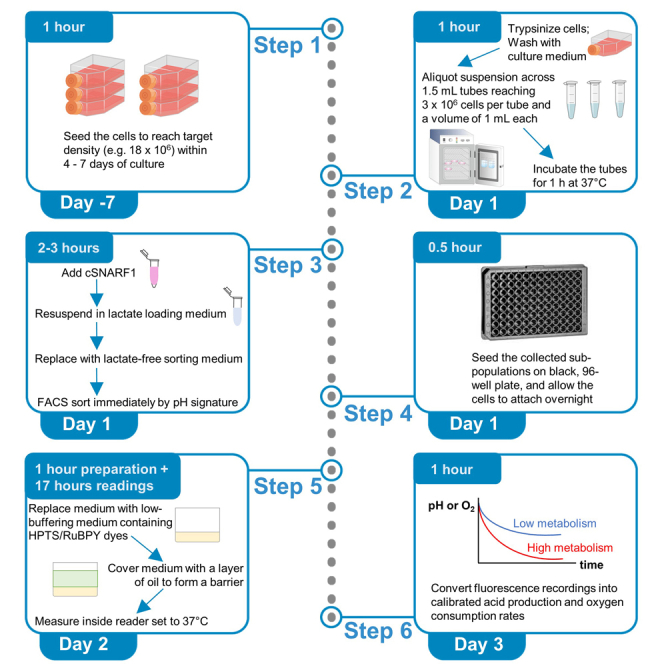

## Before you begin

A key phenotype of cancer cells is their metabolic activity, which includes a description of lactic acid fermentation and mitochondrial respiration that supply energy and building blocks for proliferation. Metabolic activity is determined by the abundance, distribution, and activity of enzymes and transporters, as well as the spatio-temporal profile of substrates, intermediates, and products. This complexity provides scope for dynamic metabolic heterogeneity which manifests in cancers, ostensibly because it may offer a growth advantage. However, mechanisms of this heterogeneity are challenging to study because metabolic rate is difficult to resolve at single-cell level, let alone use it to separate sub-populations by metabolic phenotype. Single-cell transcriptomics and proteomics can describe the network of enzymes and transporters, but fluxes are not readily inferred from this information. Single-cell metabolomics provide a snapshot of metabolite abundance at steady-state, rather than their flux.

Attempts to separate cells by metabolic activity should consider a metric related to flux that can be implemented for sorting techniques, such as FACS. Fluorescent sensors of metabolite abundance are now available for real-time measurements, but sorting by these signals separates cells by steady-state metabolite levels, which is not necessarily proportional to flux. The challenge is that conventional flow cytometry takes a single measurement per cell, which – by definition – cannot interrogate changes over time. One strategy could involve a carefully timed experiment, where a substrate is added and its intracellular abundance probed flow-cytometrically after a fixed time delay. However, this may require cells to be substrate-depleted to assign a baseline: a maneuver that may affect metabolism. Moreover, many metabolites enter into a steady-state, from which flux is not possible to calculate without an intervention, such as enzyme inhibition. A concern is whether steps required for sorting introduce stress that compromises flux estimates from metabolite abundance, a relatively labile variable.

Recently, we presented evidence^1^ that a cell’s fermentative rate relates to the membrane’s permeability to lactic acid (P_Lactic_), a process facilitated by monocarboxylate transporters (MCTs). Net lactic acid production must equal its removal across the membrane, determined from the product of P_lactic_ and the transmembrane driving force. A highly glycolytic cell benefits from higher P_lactic_ because this prevents excessive accumulation of lactate and H^+^ ions in cytoplasm. Indeed, hypoxic induction of MCT4^2^ represents an effort to match lactic acid production with efflux capacity. Since P_lactic_ is a property of the membrane, it is likely to be more stable than metabolite levels during flow cytometry protocols because internalization or trafficking are not likely to be significant during short protocols. Conveniently, lactic acid efflux across the surface membrane – irrespective of whether it is through the lipid bilayer as the undissociated acid or as H^+^-lactate co-transport by MCT – generates a pH change that can be measured using calibratable fluorescent pH dyes, such as cSNARF1, used widely for ratiometric fluorimetry. Indeed, the standard assay for P_lactic_ is to measure the rate of intracellular pH (pHi) change triggered by a maneuver that alters the driving force, typically extracellular lactate.^3^ Based on these observations, we designed a protocol that approximates P_lactic_ from the change in pHi in response to a carefully timed protocol that involves pre-equilibrating cells with lactate followed by rapid removal to drive lactic acid efflux. Cells that produce the largest alkaline transients have the highest P_lactic_. Subsequent metabolic phenotyping using our fluorimetric method^4^ confirmed that these cells produce a higher fermentative rate, alongside higher respiratory rate, indicating a state of elevated metabolic activity. Strikingly, the metabolic contrast between sorted subpopulations was short-lived, which is consistent with dynamic behavior, whereby cells alternate between metabolic state. Our finding underscores the importance of sorting cells by a surrogate of flux, and presents a simple method of achieving high contrast between emergent sub-populations for subsequent studies. As an illustration of the utility of our method, we have been able to profile subpopulations for transcriptomics in order to interrogate the underlying mechanisms of differential metabolic activity.^1^

### Preparation of media and cells for sorting by metabolic activity

**Timing: up to 1 week before sorting**

1. Prepare “lactate-loading medium”, “lactate-free sorting medium”, and “low-buffering medium” by mixing ingredients listed in recipe in materials and equipment section*.*
  a. Once dissolved, heat solution to 37°C and titrate pH to 7.4 with 4 M NaOH or 5 N HCl, as necessary.
  b. Sterile filter using a 0.22 μm filter unit.

**Pause point:** Store medium at 4°C until use.

2. Seed cells at a density determined empirically to produce at least 18 million cells in 4–7 days.
  a. Maintain cells in a standard culture medium appropriate for that line.
    ***Note:*** Information regarding the standard culture medium appropriate for a given cell line should be obtained from the cell supplier. In this protocol, we use MIA PaCa-2 cells alongside a standard culture medium of RPMI-1640 with L-glutamine and NaHCO_3_^-^, supplemented with 10% FBS, 1% penicillin-streptomycin, and 1x sodium pyruvate.
  b. Replace medium regularly to avoid excessive acidification by metabolic activity.
    ***Note:*** At least five 15 cm dishes are recommended.

### Preparation of media for metabolic phenotyping

**Timing: variable, execute ahead of experiments**

3. Prepare “low-buffering medium” by mixing ingredients listed in recipe in materials and equipment *setup.*
  ***Note:*** Minimal pH buffering enables cellular metabolism to change pH in a measurable way.
  **CRITICAL:** The recommended concentration of HEPES and MES is 2 mM, but this may be adjusted, if necessary. The HEPES-to-MES ratio should be equimolar and the [NaCl] added must be adjusted accordingly to maintain osmolarity.
  a. Once dissolved, heat solution to 37°C and titrate to the desired starting pH with 4 M NaOH or 5 N HCl, as necessary.
    ***Note:*** A starting pH of 7.4 is suggested.
    **CRITICAL:** To facilitate comparisons between experimental runs, starting pH should be adjusted with care and within 0.05 units. Only small volumes of acid/base are needed to adjust pH in low-buffer media.
  b. Sterile filter using a 0.22 μm filter unit.
    **Pause point:** Store medium at 4°C until use.
4. Prepare “high-buffering media” by mixing ingredients listed in the high-buffering medium recipe in *Materials and equipment setup.*
  ***Note:*** High pH buffering capacity ensures pH stability that is necessary for performing calibration experiments.
  a. Once dissolved, divide the solution between 8‒10 beakers.
  b. Heat solutions, one by one, to 37°C and titrate to a desired pH with 4 M NaOH or 5 N HCl, as necessary.
    ***Note:*** The range for calibration should span from pH ∼5 to ∼9 at evenly spaced intervals.
    **CRITICAL:** One calibration solution should be at pH 7.4 to represent physiological pH.
  c. Record the precise value of pH attained to 3 decimal places.
    **CRITICAL:** The precise pH values will be used to fit the calibration curve.
  d. Sterile filter using a 0.22 μm filter unit.
    **Pause point:** Store media at 4°C until use.
5. Prepare stock of fluorescent dyes:
  a. Dissolve RuBPY in sterile, deionized water (ddH_2_O) to make 100 mM stock.
  b. Dissolve HPTS in sterile, deionized water to make 4 mM stock.
  c. Mix RuBPY and HPTS stocks in a 1:1 *v/v* ratio.

**CRITICAL:** Protect fluorescent dyes from direct exposure to light.

**Pause point:** Fluorescent dye stocks can be stored for up to 2 months at −20°C.

6. Perform calibration of pH- and O_2_-sensitive dyes (Figure 1):
  **CRITICAL:** Calibrations must be performed in the same type of plate as that used for metabolic phenotyping. Consider sterile, tissue culture-treated, black wall/clear bottom plates.
  a. Under sterile conditions, thaw the HPTS/RuBPY stock mixture and dilute 1:1,000 (*v/v*) in the high-buffering media.
    **CRITICAL:** Vortex the HPTS/RuBPY stock mixture thoroughly to ensure dyes are fully dissolved. This ensures that the molar ratio of dyes is preserved.
  b. Load plate with high-buffering media containing the two fluorescent dyes.
    ***Note:*** A 96-well plate and 100 μL solution per well are recommended, using at least triplicates per calibration point.
  c. Place plate in a microplate reader with a dual gas controller at 37°C. The atmosphere should be CO_2_-free and produce regulated levels of O_2_.
    **CRITICAL:** A suitable gas regulator is required to maintain gas composition, for example an Agilent dual CO_2_ and O_2_ gas controller.
  d. Adjust O_2_ to 21% and wait for fluorescence readings to stabilize. Measure:
    i. 510 nm emission excited at 400 nm (F_1_): pH-sensitive HPTS fluorescence;
    ii. 510 nm emission excited at 460 nm (F_2_): pH-sensitive HPTS fluorescence;
    iii. 510 nm emission excited at 416 nm (F_3_): pH-insensitive HPTS fluorescence;
    iv. 620 nm emission excited at 450 nm (F_4_), O_2_-sensitive RuBPY fluorescence.
  e. Repeat measurements in Step 6(d) over a range of O_2_ levels.
    ***Note:*** Recommended O_2_ levels are 15%, 10%, 5%, 2.5%, and 1% O_2_.
  f. Export measurements and calculate two ratios that report medium pH and O_2_:
    i. When O_2_ is set to 21%, R_pH_ is defined as F_2_/F_1_.
      ***Note:*** This ratio is not expected to change with O_2_ partial pressure.
    ii. In wells at pH 7.4, R_O2_ is defined as F_3_/F_4_.
      ***Note:*** This ratio is not expected to change with pH.
  g. Obtain calibration parameters by fitting HPTS and RuBPY calibration curves:
    i. Fit the relationship between recorded pH and calculated R_pH_ to equation:
      $$\text{pH}={\text{pK}}_{a}-\log\left(\frac{r_{\max}-R_{\text{pH}}}{R_{\text{pH}}-r_{\min}}\right)$$
      where *pK*_*a*_, *r*_*max*_ and *r*_*min*_ are the calibration variables.
      ***Note:*** Curve-fitting can be performed using a package such as MATLAB or bespoke methods. Exemplar calibration curves are provided elsewhere.^4^
    ii. Normalize R_O2_ to recording at 21% O_2_ and fit the O_2_-dependence with a line constrained to cross 21% O_2_ at R_O2_ = 1:
      $$O_{2}=21\times \left(1-\left(\frac{1-R_{O2}/R_{\text{normoxia}}}{1-r_{\text{anoxia}}}\right)\right)$$
      Extrapolation of the line to 0% O_2_ estimates R_O2_ under anoxia (*r*_*anoxia*_).
      ***Note:*** Curve-fitting can be performed using a package such as MATLAB or bespoke methods. Exemplar calibration curves are provided elsewhere.^4^ A typical value for r_anoxia_ is 0.7.
      **CRITICAL:** Every set-up will have a unique calibration curve. Calibrations are not necessary after every experiment but should be performed routinely (e.g. several times a year) or after major changes in equipment, including upgrades and servicing.

**Figure 1:**
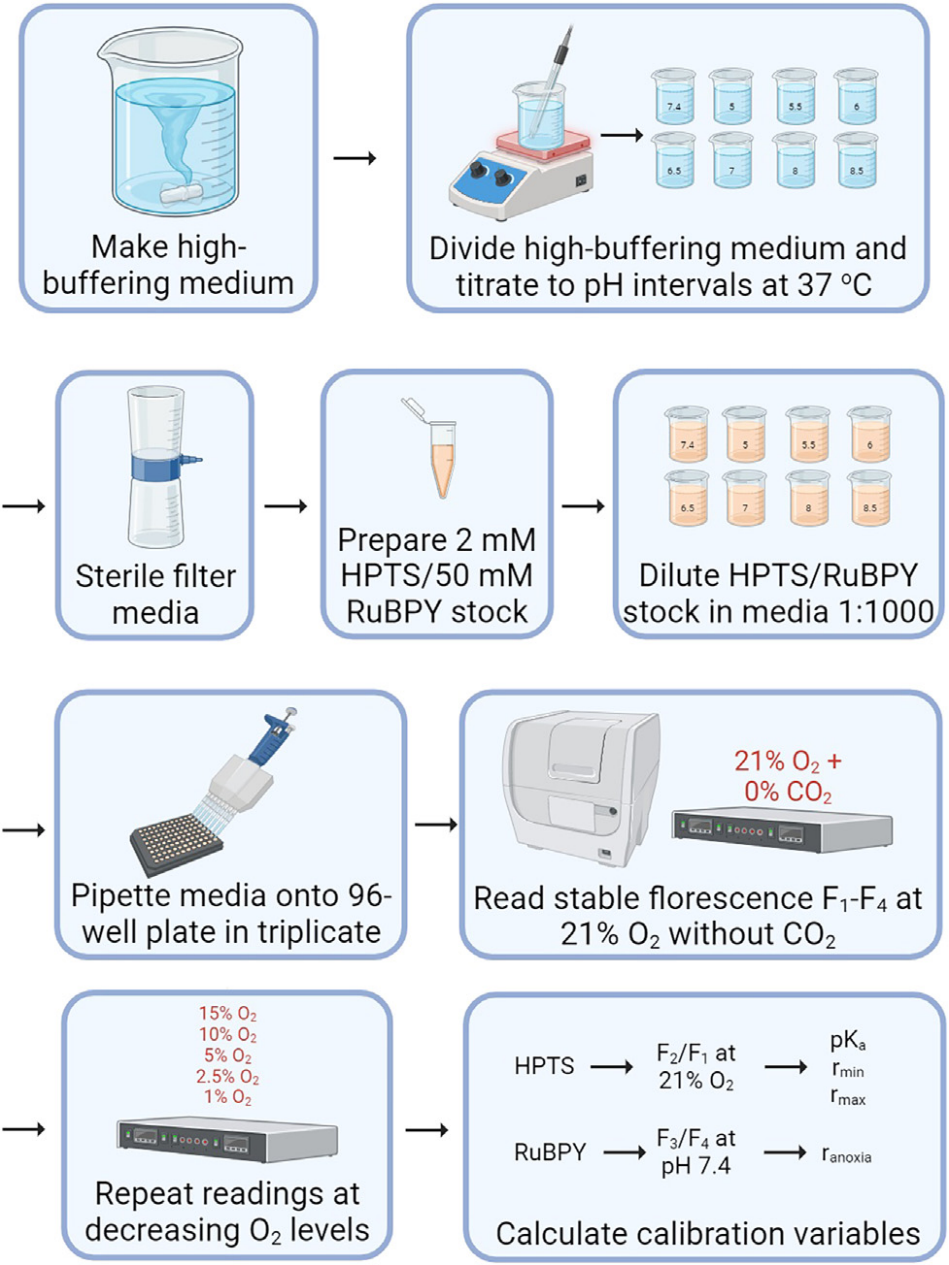
Schematic of protocol to calibrate HPTS and RuBPY fluorescence
7. Calculate the permeability of the oil barrier for measuring O_2_ consumption:
  ***Note:*** Respiratory rate is inferred from O_2_ consumption, but an open system would rapidly equilibrate medium and atmospheric O_2_. To facilitate medium O_2_ depletion, an oil barrier is placed on top of media to slow atmospheric O_2_ ingress and allow metabolism to meaningfully change dissolved O_2_. The estimate of O_2_ consumption must consider O_2_ ingress, which is a product of permeability and gradient. The former depends on the volume of oil and can be calculated by imposing a O_2_ gradient and measuring the rate of dissolved O_2_ in the medium:
  a. Load plate with fluorescent dye-containing high-buffering medium at pH 7.4.
    ***Note:*** A 96-well plate and solutions at 100 μL per well are recommended, using at least triplicates per calibration point.
  b. Gently tilt the plate and add a volume of light mineral oil over wells to control the magnitude of the O_2_ barrier.
    ***Note:*** Volumes of 0, 50, 100 and 150 μL are recommended.
    **CRITICAL:** Dispense the oil gently by touching pipette tip against well wall.
  c. Deplete O_2_ in the medium beneath the oil barrier by equilibrating the plate for at least 6 h at 37°C under a CO_2_-free atmosphere with minimal or no O_2_.
    ***Note:*** The incubator must have regulated O_2_ levels.
  d. Remove plate lid and rapidly transfer the plate to the microplate reader with dual gas controller at 37°C and maintain a CO_2_-free atmosphere with 21% O_2_. This drives O_2_ ingress down a partial pressure gradient across the oil barrier to the hypoxic medium.
    **CRITICAL:** O_2_ ingress will begin during plate transfer, therefore this step must be as fast as practical.
  e. Measure fluorescence at regular (e.g., 1–5 min) intervals, until a plateau is attained:
    i. 510 nm emission excited at 416 nm (F3): pH-insensitive HPTS fluorescence;
    ii. 620 nm emission excited at 450 nm (F_4_), O_2_-sensitive RuBPY fluorescence.
  f. Export measurements and calculate ratio R_O2_ as F_3_/F_4_. The time course of R_O2_ describes medium re-oxygenation. Best-fit to a mono-exponential curve:
    $$R_{O2}=a-b\times \exp\left(-P_{O2}\times t\right)$$
    estimates the O_2_ permeability (P_O2_) of the oil barrier. This information is used to calculate O_2_ ingress driven by oxygen-consuming cells.
    ***Note:*** Curve-fitting can be performed using a package such as MATLAB or bespoke methods.

## Key resources table

**Table:** 

REAGENT or RESOURCE	SOURCE	IDENTIFIER
**Chemicals, peptides, and recombinant proteins**		

1x PBS	Gibco	14190-094
5-(and-6)-Carboxy SNARF-1 acetoxymethyl ester	Invitrogen	C1272
8-Hydroxypyrene-1,3,6-trisulfonic acid (HPTS)	Sigma-Aldrich	H1529
Cell tracker orange CMRA	Life Technologies	C34551
D-(+)-galactose	Sigma-Aldrich	G5388
D-(+)-glucose	Sigma-Aldrich	G7021
DAPI	Sigma-Aldrich	D9542
NaHCO_3_-, glucose- and phenol red-free DMEM	Sigma-Aldrich	D5030
DMSO	Sigma-Aldrich	5895690100
EGTA	Sigma-Aldrich	324626
Fetal Bovine Serum (FBS)	Merck Life Science	F9665
GlutaMAX	Life Technologies	35050038
HEPES	Sigma-Aldrich	H3375
Hydrochloric acid	Scientific Laboratory Supplies	CHE2156
MES	Sigma-Aldrich	M3671
Mineral oil	Merck Life Science	M5904
Magnesium sulfate	Sigma-Aldrich	M7506
Penicillin-Streptomycin	Sigma-Aldrich	P0781
Potassium chloride	Sigma-Aldrich	P3911
RPMI 1640	Merck Life Science	R0883
RPMI 1640 amino acids solution	Sigma-Aldrich	R7131
RPMI 1640 vitamins solution	Sigma-Aldrich	R7256
Sodium L-lactate	Sigma-Aldrich	L7022
Sodium phosphate dibasic	Sigma-Aldrich	S9763
Sodium bicarbonate	Sigma-Aldrich	S5761
Sodium chloride	Sigma-Aldrich	S5653
Sodium hydroxide	Sigma-Aldrich	S5881
Sodium pyruvate	Gibco	11360-070
Tris(bipyridine)ruthenium(II) chloride (RuBPY)	Sigma-Aldrich	224758
Trypsin-EDTA	Gibco	15400-054

**Experimental models: Cell lines**		

Human: MIA PaCa-2	Alessandra Fiorio, University of Lille	N/A

**Software and algorithms**		

BD FACSDiva Software	BD Biosciences	https://www.bdbiosciences.com/en-us/products/software/instrument-software
analysis_script.Rmd	This paper	Supplement
Gen5 v.10	BioTek	https://www.agilent.com/en/support/biotek-software-releases
MATLAB R2020b	MathWorks	https://uk.mathworks.com/products/new_products/release2020b.html
RStudio	Posit, PBC	https://posit.co/downloads/
readxl R package	Wickham & Bryan, 2023	https://cran.r-project.org/web/packages/readxl/index.html
tidyverse R package	Wickham et al., 2019	https://cran.r-project.org/web/packages/tidyverse/index.html
writexl R package	Ooms, 2024	https://cran.r-project.org/web/packages/writexl/index.html

**Other**		

0.22 μm filter unit	Starlab	CC8221-5226
15 cm dish	VWR	734-2818
1.5 mL tube	Eppendorf	0030121023
5 mL tube with cell strainer	Falcon	352235
50 mL tube	Starlab	E1450-0100
500 mL filtering unit	Corning	10016110
Automated cell counter	Invitrogen	AMQAX1000
BD FACSAria III	BD Biosciences	N/A
BioTek Cytation 5 microplate reader	Agilent	CYT5MV
CO_2_ and O_2_ gas controller	Agilent	1210013
Incubator with O_2_ control	Scientific Laboratory Supplies	INC6376
pH meter	Mettler Toledo	30046240
Sterile, tissue culture-treated, black wall/clear bottom 96-well plate	Greiner	655077

## Materials and equipment

**Table:** Low-buffering medium (for metabolic phenotyping)

Reagent	Final concentration	Amount
DMEM: NaHCO_3_-, glucose- and Phenol Red-free	1x	4.15 *g*
HEPES	2 mM	0.238 *g*
MES	2 mM	0.195 *g*
Sodium chloride	40 mM	1.16 *g*
D-glucose	25 mM	2.25 *g*
Sodium pyruvate	1%	5 mL
Glutamax	1%	5 mL
Penicillin streptomycin	1%	5 mL
ddH_2_O	N/A	485 mL
Fetal bovine serum (FBS)	10%	50 mL

Store at 4°C for up to 4 months.

**Table:** High-buffering medium (for calibration)

Reagent	Final concentration	Amount
DMEM: NaHCO_3_-, glucose- and Phenol Red-free	1x	4.15 *g*
HEPES	10 mM	1.19 *g*
MES	10 mM	0.976 *g*
Sodium chloride	34 mM	0.993 *g*
D-glucose	25 mM	2.25 *g*
ddH_2_O	N/A	500 mL

Store at 4°C for up to 4 months.

**Table:** Lactate-loading medium (for loading cells prior to sorting)

Reagent	Final concentration	Amount
RPMI 1640 amino acids solution	1x	20 mL
RPMI 1640 vitamins solution	1x	10 mL
Potassium chloride	0.4 *g*/L	0.2 *g*
Magnesium sulfate	0.05 *g*/L	0.025 *g*
Sodium phosphate dibasic	0.8 *g*/L	0.4 *g*
HEPES	10 mM	1.19 *g*
Sodium chloride	53 mM	1.53 *g*
Sodium L-lactate	60 mM	3.36 *g*
D-glucose	11 mM	0.99 *g*
EGTA	0.5 mM	0.095 *g*
Glutamax	1%	5 mL
Sodium pyruvate	1%	5 mL
Penicillin streptomycin	1%	5 mL
ddH_2_O	N/A	455 mL

Store at 4°C for up to 4 months.

**Table:** Lactate-free sorting medium (during cell sorting)

Reagent	Final concentration	Amount
RPMI 1640 amino acids solution	1x	20 mL
RPMI 1640 vitamins solution	1x	10 mL
Potassium chloride	0.4 *g*/L	0.2 *g*
Magnesium sulfate	0.05 *g*/L	0.025 *g*
Sodium phosphate dibasic	0.8 *g*/L	0.4 *g*
HEPES	10 mM	1.19 *g*
Sodium chloride	113 mM	3.28 *g*
D-glucose	11 mM	0.99 *g*
EGTA	0.5 mM	0.095 *g*
Glutamax	1%	5 mL
Sodium pyruvate	1%	5 mL
Penicillin streptomycin	1%	5 mL
ddH_2_O	N/A	455 mL

Store at 4°C for up to 4 months.

## Step-by-step method details

### Sorting the metabolic subpopulations according to the lactate efflux capacity

**Timing: 2–3 h, depending on number of samples required**

This step separates and collects sub-populations of distinct metabolic activity, inferred from the magnitude of the intracellular pH (pHi) transient evoked upon lactate removal.

**Figure 2:**
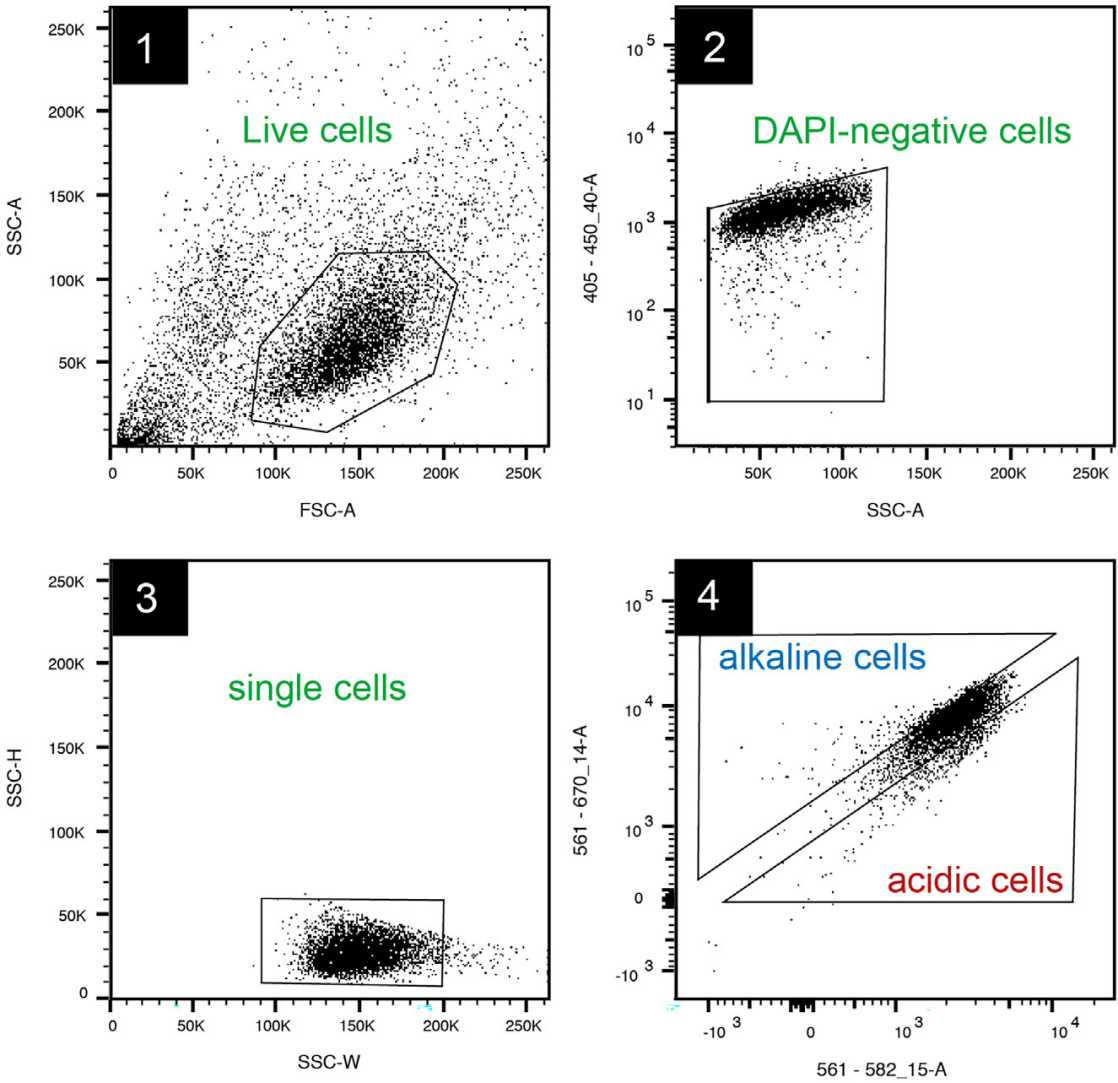
Gating strategy Step 1: Draw a gate around the bulk of the cell population. Step 2: Exclude DAPI-stained (i.e., dead) cells to ensure that only living cells are analyzed and collected. Step 3: Exclude cell doublets. Step 4: Draw a gate that represents alkaline cells (i.e., cells of high lactate efflux capacity, corresponding to higher metabolic rate) and a gate around acidic cells (i.e., cells of low lactate efflux capacity, corresponding to lower metabolic rate).
4. Sort and collect cells separated by metabolic activity:
  a. Add cSNARF1 from stock to the cell suspension to a concentration of 10 μM.
  b. Mix and incubate at room temperature (20°C–24°C) for 10 min.
  c. Centrifuge tube at 400–800 *g* at room temperature (20°C–24°C).
  d. Remove the supernatant and resuspend pellet in lactate-loading medium.
  e. Incubate for at least 5 min at room temperature (20°C–24°C).
    **CRITICAL:** The next four steps (f-i) must be performed as rapidly as practical.
  f. Centrifuge the tube at 400–800 *g* at room temperature (20°C–24°C).
  g. Remove lactate-containing supernatant and resuspend the pellet in lactate-free sorting medium.
  h. Strain through a cell-strainer.
  i. Add 1 mg/mL DAPI and run the sample through the sorter.
    ***Note:*** Preparation of the next samples for sorting can begin at this point with cSNARF1 loading.
  j. Collect cells into 1.5 mL tubes.
  k. Replace with new tubes when cell count reaches 2 × 10^5^ cells.
    **CRITICAL:** Note the exact number of cells collected.
  l. Repeat steps a-k for the remaining samples.
5. Optional: Seed the collected cells for metabolic phenotyping:
  a. Spin down the collected cells at 600 *g* for 5 min at room temperature (20°C–24°C).
  b. Gently aspirate the supernatant and resuspend cells in fresh culture medium, as appropriate for the cell type under investigation.
  c. Seed collected cells in equal numbers onto separate wells as soon as possible.

***Note:*** Best results are achieved with seeding densities 30,000 to 80,000 cells/well.

***Note:*** Sterile, tissue culture-treated, black wall/clear bottom 96-well plates are recommended. If the cell yield is insufficient, 384-well plates with smaller wells can be used.

1. Prepare cells on the day of sorting:
  a. Obtain culture vessels (e.g., plates) containing cultured cells.
  b. Aspirate media and wash with 1x phosphate-buffered saline (PBS).
  c. Add sufficient volume of 2x Trypsin-EDTA mixture to cover the plate and incubate at 37°C for 3 min until cells detach.
    ***Note:*** This step is omitted when using non-adherent cells.
  d. Collect floating cells into 50 mL tube by washing the plate with fresh culture medium.
  e. Count the collected cells using hemocytometer or automated cell counter.
  f. Centrifuge cells at 400–600 *g* for 5 min at room temperature (20°C–24°C).
  g. Discard the supernatant and resuspend the pellet in standard culture medium, as deemed appropriate for the cell line of choice.
    ***Note:*** The recommended target density is 3 × 10^6^ cells/mL.
  h. Aliquot the cell suspension across 1.5 mL sterile tubes. Set aside up to 5 additional tubes with lower cell concentrations for optimizing the gating protocol.
    ***Note:*** Consider 15–20 tubes for a yield of ∼1 × 10^5^ cells collected during 2–3 h of sorting.
  i. Place the tubes containing cells in an incubator at 37°C for up to 1 h prior to sorting.
  j. Prepare 1.5 mL cell-free collection tubes containing 500 μL of culture medium.
  k. Warm lactate-loading medium and lactate-free sorting medium and transfer sufficient volume into 50 mL tubes.
    ***Note:*** A total of 18 tubes containing 3 × 10^6^ cells each will require ∼25 mL per medium.
2. Prepare equipment for cell sorting:
  a. Follow the device manufacturer’s recommended start-up procedure.
    ***Note:*** The current protocol was optimized for a 0.85 μm nozzle and sheath pressure of 45 psi on a BD FACSAria III cell sorter.
  b. Set-up the protocol with relevant parameters:
    i. forward- and side-scatter (FSC and SSC),
    ii. DAPI (nuclear dye) fluorescence,
    iii. cSNARF1 fluorescence: recommended laser excitation is 488 nm, 514 nm or 555 nm excitation, and bandpass filters centered a 580 nm and 640 nm.
      ***Note:*** DAPI has low membrane permeability and stains nuclei of dead cells only.
      **CRITICAL:** Other pH dyes are possible, provided they are dual-emission.
  c. Position the collection tubes and pre-set their temperature to 37°C.
  d. Pre-set the temperature of the sample injection chamber to 20°C–24°C.
    ***Note:*** This temperature setting was determined empirically to reduce cell aggregation in MIA PaCa-2 cells but may need cell line-specific optimization.
    **CRITICAL:** Avoid lower temperatures as this may have negative effects on metabolism.
3. Optimize gating settings (see also Figure 2 for the recommended gating strategy):
  a. Define gates for excluding dead cells and cell doublets:
    i. Add 1 mg/mL DAPI to the test sample and run through the sorter.
    ii. Draw a gate on the FSC-area/SSC-area scatterplot that captures the bulk of cells.
    iii. Use the DAPI channel to exclude dead cells that stain with DAPI.
    iv. Exclude cell doublets using the SSC-height/SSC-width scatterplot.
  b. Define the cSNARF1 fluorescence gate that represents the most acidic cells:
    i. Add cSNARF1 from stock to ∼1 mL cell suspension to a final concentration of 10 μM and incubate at room temperature (20°C–24°C) for 10 min.
      ***Note:*** cSNARF1 stock is prepared by dissolving 50 μg of the dye with 50 μL DMSO.
    ii. Centrifuge at 400–800 *g* at room temperature (20°C–24°C).
    iii. Remove the supernatant and resuspend the pellet in 1 mL lactate-loading medium.
    iv. Incubate for at least 5 min at room temperature (20°C–24°C) to allow for lactate equilibration across the cell membrane.
    v. Remove debris and cell aggregates by pipetting sample through cell strainer cap tube.
    vi. Add 1 mg/mL DAPI and run the sample on the sorter using gates defined in Step 10(a).
    vii. On the pair of cSNARF1 fluorescence channels, draw a gate around the emergent population.
      ***Note:*** This region corresponds to cells of the lowest metabolic activity.
  c. Define the cSNARF1 gate that represents cells with the largest alkaline transients.
    i. Add cSNARF1 from stock to ∼1 mL cell suspension to a concentration of 10 μM and incubate at room temperature (20°C–24°C) for 10 min.
    ii. Centrifuge at 400–800 *g* at room temperature (20°C–24°C).
    iii. Remove the supernatant and resuspend the pellet in lactate-loading medium.
    iv. Incubate for at least 5 min at room temperature (20°C–24°C) to allow for lactate equilibration across the cell membrane.
      **CRITICAL:** The next four steps (v-viii) must be performed as rapidly as practical.
    v. Centrifuge at 400–800 *g* at room temperature (20°C–24°C).
    vi. Remove lactate-containing supernatant and resuspend the pellet in 1 mL lactate-free sorting medium.
    vii. Pass sample through cell-strainer.
    viii. Add 1 mg/mL DAPI and run the sample on the sorter using gates defined in Step 10(a).
    ix. Record events as soon as possible and at regular intervals thereafter. Cells producing the largest alkaline transients will have a higher 640/590 fluorescence ratio.
      ***Note:*** Time points of 2, 5, 7, 10 and 12 min are recommended but may require optimizing in a cell line-specific manner.
    x. Draw a gate around the most prominent alkaline population.
      ***Note:*** This region corresponds to cells of the highest metabolic activity.
      **CRITICAL:** The proportion of cells falling into this region should not exceed 20%.

**CRITICAL:** Handle cells as soon as possible to avoid cell death. If a delay is unavoidable, place tubes with collected cells in the incubator.

**CRITICAL:** When seeding cells, reserve the outermost wells of the plate for filling with PBS to reduce evaporation from innermost wells of the plate.

**CRITICAL:** Include wells with medium but no cells to represent cell-free controls.

### Metabolic phenotyping of sorted subpopulations

**Timing: several hours, typically overnight, followed by off-line analysis time. Here: 1 h to prepare the plate for measurements, 17 h of fluorescence readings, 1 h of analysis.**

Metabolic phenotyping uses a fluorimetric method to simultaneously measure medium pH and dissolved O_2_. This approach can be used to confirm sorting of cells by metabolic rate. Alternatively, the method can be used as a stand-alone assay.

6. Obtain media with dissolved pH- and O_2_-sensitive fluorescent dyes:
  a. Warm low-buffering medium to 37°C and thaw an aliquot of the HPTS/RuBPY stock.
    **CRITICAL:** Thoroughly vortex the HPTS/RuBPY stock mixture to ensure dyes are completely dissolved and evenly mixed.
  b. Dilute the HPTS/RuBPY stock mixture 1:1,000 (*v/v*) in low-buffering medium.
7. Load 96-well (or similar) plate under sterile conditions:
  a. Once cells have become adherent, remove the plate from the incubator.
    **CRITICAL:** Include cell-free wells to obtain a baseline for referencing metabolic activity.
  b. Replace medium with 100 μL of fluorescent dye-containing low-buffering medium.
  c. Gently tilt the plate and dispense 150 μL of mineral oil over the wells by touching the upper walls whilst discharging the pipette.
    ***Note:*** The oil is a barrier to O_2_ ingress, which is necessary for enabling respiration to meaningfully reduce dissolved O_2_. This barrier will also reduce CO_2_ egress and contribute to medium acidification.
    ***Note:*** Some wells can be left without an oil barrier to report acidification rate due to fermentation, without a component due to CO_2_ hydration.
8. Perform fluorescence measurements:
  a. Place plate (with lid) in the microplate reader at 37°C in a CO_2_-free atmosphere.
  b. Record fluorescence at regular (e.g., 10 min) intervals:
    i. 510 nm emission excited at 400 nm (F_1_): pH-sensitive HPTS fluorescence;
    ii. 510 nm emission excited at 460 nm (F_2_): pH-sensitive HPTS fluorescence;
    iii. 510 nm emission excited at 416 nm (F_3_): pH-insensitive HPTS fluorescence;
    iv. 620 nm emission excited at 450 nm (F_4_), O_2_-sensitive RuBPY fluorescence.
      ***Note:*** The duration of measurements can be as long as required: 18 h is recommended.
  c. Export measurements and calculate R_pH_ as F_2_/F_1_ and R_O2_ as F_3_/F_4_.
9. Run the analysis:
  a. Export the data into a workbook (e.g., Excel). See Data S1 for example.
    **CRITICAL:** Implement one channel per sheet, with columns corresponding to wells and rows corresponding to time.
  b. Prune columns that relate to empty or PBS-containing wells.
  c. Format the workbook using the example workbook as a template.
    **CRITICAL:** To expedite analysis, ensure all sheet names and column headings in the “sample_ metadata” sheet match (including case) those in example workbook. Columns should begin from A1 and there should be no gaps between rows or columns. In the sheets corresponding to channels (HPTS_400, HPTS_460, HPTS_isosbestic, and RuBPY), the first column should be time in the format “hh:mm:ss”. In the sample metadata sheet, cell-free controls should be labeled as “blank” under the subpopulation column.
10. Run the analysis script in RStudio using code provided in Code S2.
  a. Open RStudio and create a new R Project directory.
  b. Within the directory, save a copy of analysis_script.Rmd and the formatted workbook.
    ***Note:*** If necessary, install the packages listed in Section 1 of the analysis script according to system dependencies: tidyverse, readxl, and writexl.
  c. Update the calculated HPTS and RuBPY calibration variables to Section 2 of the analysis script.
    **CRITICAL:** The calibrations given in the scripts are exemplar and must be replaced with measured values applicable to the equipment used.
    ## HPTS ##
    # pKa
    pKa <- 7.5284
    # rmax
    rmax <- 4.6038
    # rmin
    rmin <- 0.0420
    ## RuBPY ##
    #ranoxia
    ranoxia <- 0.7125
  d. Update the constants specific to the experimental setup in Section 3 of the analysis script:
    i. volume of medium/well in mL (V),
    ii. calculated oxygen permeability constant (P_O2_).
      # volume of medium/well (mL)
      V <- 0.1
      # calculated oxygen permeability constant (min-1)
      PO2 <- 0.029
  e. Amend the file paths to the input Excel workbook in Section 4 of the analysis script.
    # channel 1 data
    hpts_400 <- read_excel("/path/to/your/workbook.xlsx", sheet="HPTS_400")
    # channel 2 data
    hpts_460 <- read_excel("/path/to/your/workbook.xlsx", sheet="HPTS_460")
    # channel 3 data
    hpts_iso <- read_excel("/path/to/your/workbook.xlsx", sheet="HPTS_isosbestic")
    # channel 4 data
    ru <- read_excel("/path/to/your/workbook.xlsx", sheet="RuBPY")
    # well annotations
    sample_metadata <- read_excel("/path/to/your/workbook.xlsx", sheet="sample_metadata")
  f. Amend the file paths to the output Excel files and plots in Section 10 of the analysis script.
    ## pH data ##
    # save the summary data as an Excel file
    write_xlsx(summarised_pH,
      "/path/to/your/pH_data.xlsx")
    ## cumulative acid production data ##
    # save the summary data as an Excel file
    write_xlsx(summarised_acid_prod,
      "/path/to/your/acid_prod_data.xlsx")
    ## O2 data ##
    # save the summary data as an Excel file
    write_xlsx(summarised_O2,
      "/path/to/your/O2_data.xlsx")
    ## cumulative oxygen consumption data ##
    # save the summary data as an Excel file
    write_xlsx(summarised_O2_consum,
      "/path/to/your/O2_consum_data.xlsx")
  g. Run all sections sequentially.

## Expected outcomes

**Figure 3:**
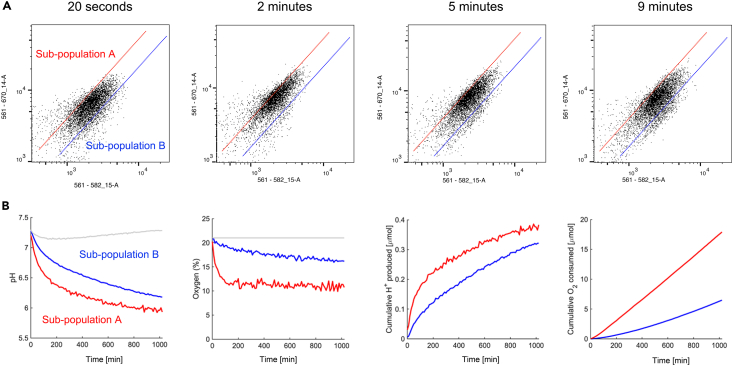
Exemplary data depicting metabolic heterogeneity of the MIA PaCa-2 cell line Panel A shows a series of scatter plots of pH in cells during evoked lactate efflux. The alkaline population (red; A) corresponds to the sub-population of cells with higher lactate efflux capacity, while the acidic sub-population (blue; B) describes cells with lower lactate efflux capacity. Note, the transient character of the alkaline sub-population. Panel B depicts the distinct metabolic phenotypes of collected subpopulations in terms of medium pH and medium oxygen, cumulative acid production and oxygen consumption. For clarity, a single representative repeat is shown.

The sorting protocol separates cells by capacity to remove lactic acid, as determined by the pH signature of cells during an outward lactic acid gradient. According to our finding,^1^ cells with higher lactate efflux capacity are associated with higher metabolic rate, both fermentative and respiratory, as confirmed using the metabolic phenotyping. The membrane impermeable ratiometric pH-dye HPTS tracks medium pH which provides a readout of metabolic rate, most of which is due to lactic acid production, a non-volatile acid. The rate of acid production is calculated from the product of pH change (measured during the experiment) and buffering capacity (measured separately, in cell-free experiments). Cumulative acid production is calculated from the sum of acid-production flux over time. RuBPY, normalized to the isosbestic (i.e., pH-insensitive) HPTS wavelength, provides a measure of dissolved oxygen. The rate of O_2_ consumption by cells must consider the change in dissolved O_2_ plus the rate of O_2_ ingress from the atmosphere. The latter is calculated from the difference in O_2_ between the medium and atmosphere multiplied by the O_2_ permeability of the oil barrier. Following metabolic verification that the sub-populations are distinct, further experiments can be performed to understand the basis of metabolic heterogeneity. Exemplary data showing the time-course of transient populations collected by FACS and phenotyping data obtained with described protocol are shown in Figure 3.

## Quantification and statistical analysis

The analysis of pH and O_2_ time courses converts raw fluorescence reads into measurements of metabolic acid production and respiratory O_2_ consumption, respectively. The computational pipeline for processing these data is provided in the R markdown file analysis_script.Rmd and may be run in RStudio. The main input required is a workbook which may be exported from a plate reader, and formatted using the example_workbook.xlsx as a template. In addition, calculated calibration variables and setup-specific constants must be updated within the script for accurate analyses. The main outputs are Excel files providing summary statistics of each time interval and sorted subpopulation, covering:

• medium pH,
• cumulative acid production (μmol),
• medium oxygen (%),
• cumulative oxygen consumption (μmol).

Examples of these outputs generated by running the analysis for example_workbook.xlsx are provided in the supplemental information.

Medium pH is derived from the ratio of deprotonated to protonated HPTS (R_pH_) following Equation 1.

(Equation 1) $$\text{pH}={\text{pK}}_{a}-\log\;\left(\frac{r_{\max}-R_{\text{pH}}}{R_{\text{pH}}-r_{\min}}\right)$$

**Figure 4:**
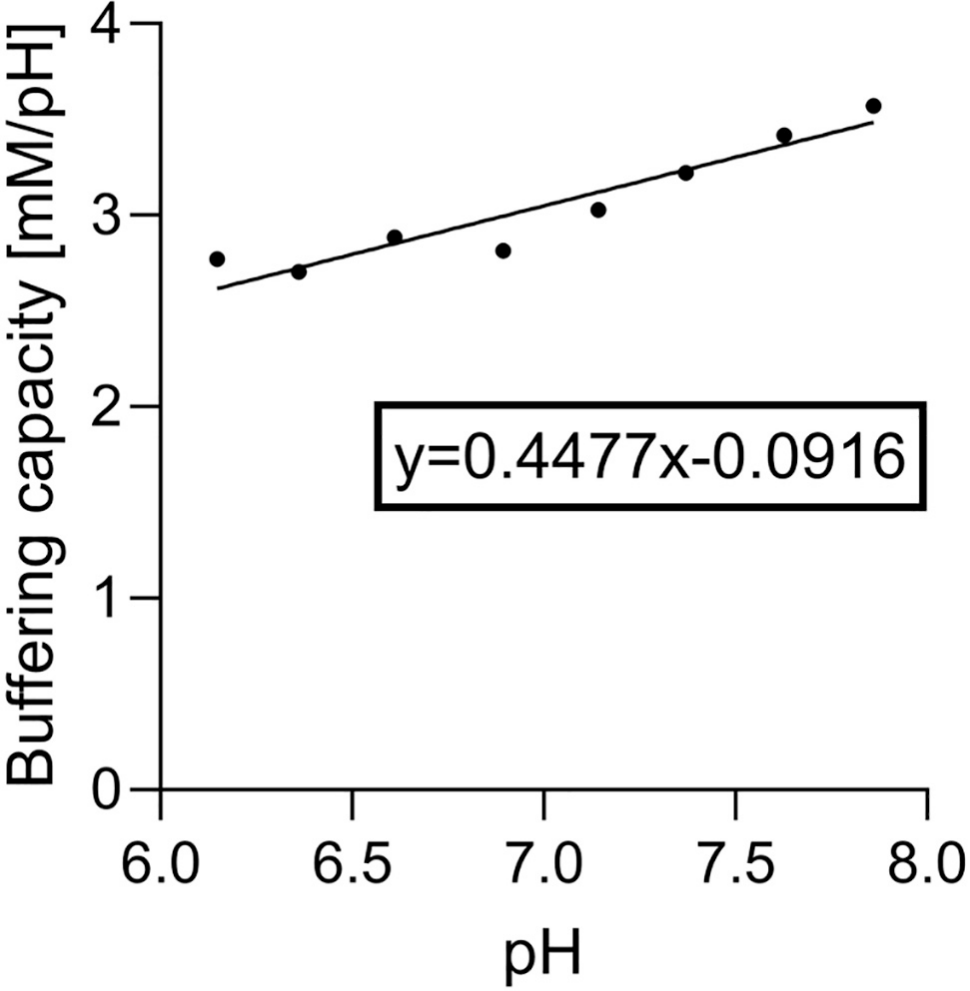
The pH-sensitivity of buffering capacity of the low buffering medium

Changes in medium pH reported by HPTS represent the levels of free H^+^ ions in the medium. However, chemical systems manifest pH buffering attributable to serum proteins and buffering agents such as HEPES and MES. Consequently, many H^+^ ions generated by metabolism will be buffered. To describe the *total* metabolic H^+^ flux, buffering must be accounted for using its quantitative measure, β. Thus, the flux of H^+^ ions produced (J^H^) is the product of the negative pH change (dpH/dt) and buffering capacity β. The sign is inverted because acid production reduces pH. Buffering is a function of medium pH, which we have measured previously for low-buffering medium containing 2 mM equimolar HEPES:MES (Figure 4). Cumulative acid production is calculated from the sum of H^+^ fluxes (J_H_) over time (from 0 to T) in cell-containing wells above cell-free controls. To convert concentration into molar amount, production is multiplied by medium volume (V), as shown in Equation 2.

(Equation 2) $$C_{H}=V\times \left(-\sum_{t=0}^{T}\left(\Delta pH^{\text{cells}}\times \beta \right)+\sum_{t=0}^{T}\left(\Delta pH^{\text{cell}-\text{free}}\times \beta \right)\right)$$

RuBPY fluorescence, as measured by F_4_, is quenched by oxygen. To provide a robust readout of medium oxygen, RuBPY is expressed relative to the pH-insensitive isosbestic point of HPTS fluorescence measured as F_3_. This exploits the fixed mixing ratio. The resulting ratio R_O2_ is converted into medium oxygen, in units of % of oxygen gas, as per Equation 3 using the RuBPY calibration variable r_normoxia_.

(Equation 3) $$O_{2}=21\times \left(1-\left(\frac{1-R_{O2}/R_{normoxia}}{1-r_{anoxia}}\right)\right)$$

*In vivo*, oxygen buffering can involve chelators, such as hemoglobin. However, oxygen buffering in media is close to zero and can be ignored. Oxygen is volatile and therefore two fluxes must be considered when measuring consumption: (i) depletion of medium O_2_ and (ii) ingress of O_2_ into medium from the atmosphere. The latter is reduced by the mineral oil barrier. To convert O_2_ levels expressed as a percentage of gases (i.e., fractional partial pressure) into molar concentration, recordings are multiplied by solubility a, which can be obtained from recordings in water or saline^4^ at the appropriate temperature, taken here as 10.6 μM per %. Cumulative O_2_ consumption is calculated using Equation 4:

(Equation 4) $$C_{O2}=\alpha \times V\times (\sum_{t=0}^{T}\left(-\Delta {O_{2}}^{\text{cells}}+P_{O2}\times \text{dt}\times \left({O_{2}}^{\text{atmoshere}}-{O_{2}}^{\text{cells}}\right)\right)$$

Cell-free wells, which maintain 21% O_2_, can be used to offset measurements, if necessary.

## Limitations

The sorting protocol may not be suitable for cell lines that have insufficient metabolic heterogeneity or low population-averaged metabolic rate. The protocol yields a relatively low number of cells (<100,000) per sorting session which may not be sufficient for some types of measurements downstream. It is, however, possible to pool equivalent subpopulations from several rounds of sorting. The experimental protocol was developed on a BD FACSAria III Cell Sorter and metabolism was measured on a BioTek Cytation 5 plate reader; other equivalent systems may warrant extensive optimization.

## Troubleshooting

### Problem 1

Cell aggregates and/or debris clog the sorter: related to Steps 3 and 4.

### Potential solution

Consider running a larger number of sample tubes containing a reduced concentration of cells in suspension. Consider reducing flow rate. Ensure the sample is vortexed and strained before running through the sorter. Maintaining the temperature of the sample chamber near 20°C–24°C or an otherwise optimized temperature.

### Problem 2

An inadequate yield of cells is collected during sorting: related to Steps 3 and 4.

### Potential solution

Increase the cell concentration per tube in the sample used for sorting. Concentration can be raised to 10 million per mL without excessive aggregation, but higher concentrations carry a risk of aggregation and clogging the sorted. If the cell yield continues to be inadequate, consider adapting the subsequent protocols for smaller cell counts, such as using a 384-well plate instead of a 96-well plate.

### Problem 3

The cells collected fail to attach to the base of the 96-well plate and most cells are inadvertently lost prior to metabolic phenotyping: related to Step 5(c).

### Potential solution

This issue may arise when the time delay between cell collection and seeding is long, leading to cell death. To address this, the cells should be seeded as soon as possible after sorting. Prior to seeding, the cells should be centrifuged to visually confirm a pellet of live cells. Excessively long sorting sessions (>>3 h) should be avoided. The 96-well plate can be coated with poly-L-lysine prior to seeding to increase adhesiveness.

### Problem 4

Sub-populations separate as distinct during sorting, but no difference in metabolic phenotype emerges when assayed with the fluorimetric method: related to Step 8.

### Potential solution

This issue may arise because of a prolonged sorting window or inadequately separated gating regions. The sorting window should be limited to several minutes (<15 min) and the gates should be drawn to collect cells of distinctly separate pHi i.e., ∼20% quantiles representing the most alkaline and most acidic cells.

### Problem 5

No meaningful changes in medium pH and/or dissolved oxygen are measured: related to Step 8.

### Potential solution

For the assay to be successful, cells must collectively produce enough lactic acid or consume enough oxygen to cause measurable changes in pH and dissolved oxygen. A lack of change in medium pH or oxygen tension may represent a biologically meaningful result if the investigated cell (sub)population has very low fermentative and respiratory rates. However, if all sorted subpopulations exhibit no change in medium pH and/or oxygen tension during an overnight run, differences between sorted subpopulations cannot be resolved. To address this, cell seeding density should be increased or medium volume decreased.

## Resource availability

### Lead contact

Further information and requests for resources and reagents should be directed to and will be fulfilled by the lead contact Pawel Swietach (pawel.swietach@dpag.ox.ac.uk).

### Technical contact

Technical questions on executing this protocol should be directed to and will be answered by the lead contact.

### Materials availability

This study did not generate unique reagents.

### Data and code availability

The code to analyze the metabolic phenotyping data has been deposited. This paper does not contain any standardized datasets. All data reported in this paper will be shared by the lead contact upon request.

## Supplemental information

Data S1. Example worksheet with metabolic phenotypic data generated in Step 9 and used for processing by the R script

Code S2. Script written R for analyzing raw metabolic phenotyping data in Step 10

## References

[1] Blaszczak W., White B., Monterisi S., Swietach P. (2024). Dynamic IL-6R/STAT3 signaling leads to heterogeneity of metabolic phenotype in pancreatic ductal adenocarcinoma cells. Cell Rep..

[2] Ullah M.S., Davies A.J., Halestrap A.P. (2006). The plasma membrane lactate transporter MCT4, but not MCT1, is up-regulated by hypoxia through a HIF-1alpha-dependent mechanism. J. Biol. Chem..

[3] Blaszczak W., Williams H., Swietach P. (2022). Autoregulation of H(+)/lactate efflux prevents monocarboxylate transport (MCT) inhibitors from reducing glycolytic lactic acid production. Br. J. Cancer.

[4] Blaszczak W., Tan Z., Swietach P. (2021). Cost-Effective Real-Time Metabolic Profiling of Cancer Cell Lines for Plate-Based Assays. Chemosensors.

